# Genome-wide association study of smoking trajectory and meta-analysis of smoking status in 842,000 individuals

**DOI:** 10.1038/s41467-020-18489-3

**Published:** 2020-10-20

**Authors:** Ke Xu, Boyang Li, Kathleen A. McGinnis, Rachel Vickers-Smith, Cecilia Dao, Ning Sun, Rachel L. Kember, Hang Zhou, William C. Becker, Joel Gelernter, Henry R. Kranzler, Hongyu Zhao, Amy C. Justice, VA Million Veteran Program

**Affiliations:** 1 Yale School of Medicine, New Haven, CT 06511 USA; 2 VA Connecticut Healthcare System, West Haven, CT 06516 USA; 3 Yale School of Public Health, New Haven, CT 06511 USA; 4 University of Kentucky College of Public Health, Lexington, KY 40536 USA; 5 University of Pennsylvania Perelman School of Medicine, Philadelphia, PA 19104 USA; 6 Crescenz Veterans Affairs Medical Center, Philadelphia, PA 19104 USA

**Keywords:** Genetic predisposition to disease, Predictive markers, Risk factors

## Abstract

Here we report a large genome-wide association study (GWAS) for longitudinal smoking phenotypes in 286,118 individuals from the Million Veteran Program (MVP) where we identified 18 loci for smoking trajectory of current versus never in European Americans, one locus in African Americans, and one in Hispanic Americans. Functional annotations prioritized several dozen genes where significant loci co-localized with either expression quantitative trait loci or chromatin interactions. The smoking trajectories were genetically correlated with 209 complex traits, for 33 of which smoking was either a causal or a consequential factor. We also performed European-ancestry meta-analyses for smoking status in the MVP and GWAS & Sequencing Consortium of Alcohol and Nicotine use (GSCAN) (N_total_ = 842,717) and identified 99 loci for smoking initiation and 13 loci for smoking cessation. Overall, this large GWAS of longitudinal smoking phenotype in multiple populations, combined with a meta-GWAS for smoking status, adds new insights into the genetic vulnerability for smoking behavior.

## Abstract (web summary)

Genome-wide association studies (GWASs) for cigarette smoking have identified several hundred loci that account for a small proportion of the overall genetic risk. Here, the authors report a large GWAS for smoking trajectories and meta-analysis for smoking status, finding multiple plausible loci.

## Introduction

Cigarette smoking has an estimated heritability of 40–70%^1,2^, and is a leading cause of morbidity and mortality worldwide. In the past two decades, over 25 genome-wide association studies (GWAS) of smoking and smoking-related phenotypes have been reported^3–9^. Among several hundred reported loci, the established loci are predominantly in a few genomic regions, such as *15q25* (*CHRNA5-A3-B4*) and *8p11* (*CHRNB3-CHRNA6*)^3,4,10,11^. Large meta-GWASs have been reported, including a study from the UK Biobank (UKBB) and the Tobacco and Genetics Consortium (TAG) with a total of 518,633 individuals that identified 223 loci for ever versus (vs.) never smokers^10^. These genetic variants accounted for 10.9% of the phenotypic variation for this single trait^10^. The largest meta-GWAS to date by the GWAS & Sequencing Consortium of Alcohol and Nicotine use (GSCAN) included up to a total of 1.2 million individuals (depending on traits) from 26 cohorts and identified 406 loci associated with multiple stages of cigarette use (initiation, cessation, and heaviness)^5^. In that study, genetic heritability was 4–8% for smoking phenotypes^5^. A meta-analysis GWAS of up to 61 studies identified 40 new rare or low-frequency variants associated with smoking behavior^6^. However, none of the large GWAS studies identified genetic variants for smoking trajectories.

Smoking is a complex trait typically ascertained by self-reported responses to questionnaires. Phenotypes previously used for GWAS include self-reported ever vs. never smoked^3,12–14^, number of cigarettes smoked per day (CPD)^3,9,14^, age of initiation, smoking cessation, and nicotine dependence defined by the Fagerström Test for Nicotine Dependence (FTND)^7,15^. However, single measures of self-reported health behaviors, especially those that are stigmatized like smoking, are subject to social desirability bias and substantially underestimate smoking^16^, and they often measure state rather than trait. Biomarkers for nicotine exposure offer less biased metrics and have demonstrated strong associations with genetic markers^17,18^. For example, the nicotine metabolite ratio, which can be estimated in blood, provided an estimated heritability as high as 0.81 and was linked to *19q13*^19,20^. However, the feasibility of using biomarkers for large-scale gene discovery is limited. In contrast, electronic medical records (EMRs) provide an opportunity to obtain large-scale longitudinal data that can be linked to genetic data. The large scope of EMR-derived data increases power, overcoming some of the limitations of state phenotypic measurement. Few studies have applied a longitudinal smoking phenotype for gene discovery^21^.

**Fig. 1:**
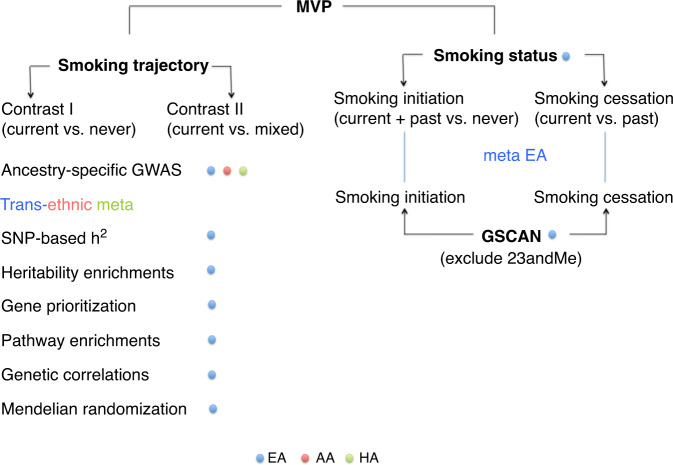
An overview of analyses performed on the smoking trajectory and status phenotypes. MVP the Million Veteran Program; GSCAN the GWAS & Sequencing Consortium of Alcohol and Nicotine use; EA European American; AA African American; HA Hispanic American; GWAS genome-wide association study; *h*^2^ heritability.

We leverage the EMR-derived longitudinal data to perform the largest longitudinal smoking GWAS in a multi-ethnic cohort and identify 16 genetic loci associated with smoking trajectory contrasts for European American (EA), one locus for African American (AA), and one locus for Hispanic American (HA) individuals from the Million Veteran Program (MVP, *N* = 286,118). We also meta-analyze smoking status GWASs from EAs in the MVP and European subjects in GSCAN (excluding data from 23andMe which summary statistics are not released for public), yielding a total sample of 842,717 individuals and leading to the identification of 99 genetic loci associated with smoking initiation and 13 loci associated with smoking cessation. We further characterize the genetic risk for smoking trajectory by estimating SNP-based heritability, prioritizing causal genes and biological pathway interpretation, determining genetic correlation of smoking trajectory with psychiatric and nonpsychiatric traits, and exploring the causality of smoking trajectory with other genetically correlated traits. The phenotypic characterization of the studied populations is presented in Table 1 and analytical approach is presented in Fig. 1. Overall, we identify multiple genetic loci associated with smoking trajectory contrasts in diverse populations. Our findings highlight the significance of incorporating longitudinal data in the EMR-derived phenotypes in improving the identification and biological interpretation of genetic associations characterizing smoking behaviors for diverse populations.

**Table 1:** Phenotype distribution of smoking trajectories and demographic characteristics in the Million Veteran Program (*N* = 286,118).

Population	Mostly current	Mixed	Mostly never	Total	Age: mean (SD)	Male (%)
EA	40,456 (19%)	110,403 (53%)	59,056 (28%)	209,915	63.8 (13.1)	93.0
AA	13,511 (25%)	23,605 (43%)	17,751 (32%)	54,867	57.7 (11.9)	87.4
HA	2920 (14%)	11,221 (52%)	7195 (34%)	21,336	55.6 (15.1)	92.0

*EA* European American, *AA* African American, *HA* Hispanic American, *SD* standard deviation.

## Results

### GWAS for EMR-based smoking trajectories in the MVP

Using a previously validated approach^22^ and data from over 2.2 million clinical encounters (per person mean = 10.5, median = 8) where smoking status was recorded in the EMR for a total of 286,118 veterans (209,915 EAs, 54,867 AAs, and 21,336 HAs), we identified three distinct longitudinal trajectory groups for smoking in each population group. The trajectory groups were defined using the highest assignment probabilities and were identified as individuals who mostly never smoked, those with mixed smoking and nonsmoking and those who, over time, reported mostly current smoking. As a binary comparison phenotype, we also defined smoking status as smoking initiation (ever vs. never) and smoking cessation (current vs. past) using a classification method based on the modal value, i.e., the most common value of all data available in the same samples. Although the two phenotypes were highly correlated, by comparing their performance we evaluated their relative utility for detecting genetic variants contributing to smoking behavior.

We first conducted a GWAS for smoking trajectories separately in EA, AA, and HA populations in the MVP, then conducted a trans-ethnic meta-GWAS for smoking trajectories. We used a multinomial regression model to analyze the unordered multinomial smoking trajectory groups and adjusted for age, sex, and ten principal components (PCs) as covariates. The pairwise smoking trajectory contrasts are current vs. never (contrast I), which corresponds to smoking initiation, and current vs. mixed (contrast II), which corresponds to smoking cessation. The two trajectory contrasts were modeled simultaneously, resulting in smaller standard error estimates of genetic effects and thus greater statistical power. The overall fit of the model was evaluated using the likelihood ratio test.

**Fig. 2:**
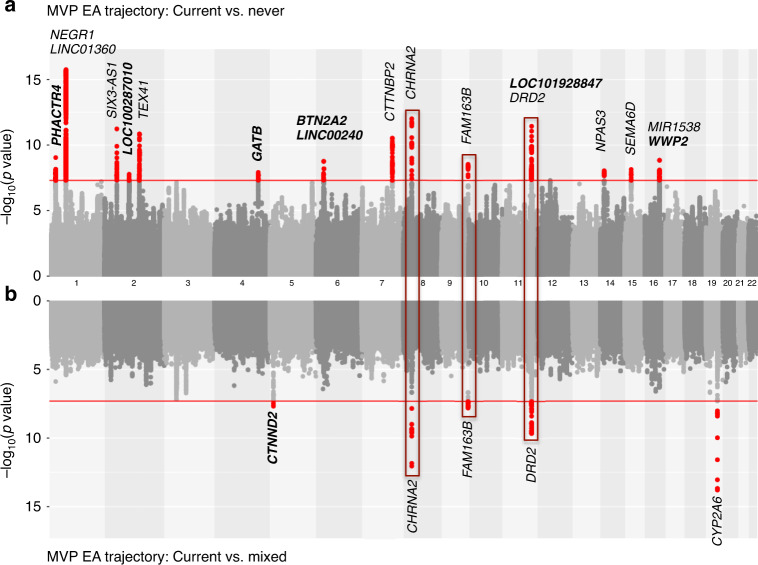
Genome-wide association study results for smoking trajectory contrasts. Mirror Manhattan plots for smoking trajectory contrast I (current vs. never (**a**)) and contrast II (current vs. mixed (**b**)) in European American samples in the Million Veteran Program. Genome-wide significant loci are highlighted in red and mapped to genes by the nearest location. Genes have not been previously reported to be associated with smoking phenotypes in other studies are marked in bold. Three loci shared by contrast I (current vs. never) and contrast II (current vs. mixed) are highlighted in red boxes.

In the EA samples, we identified 16 independent genome-wide significant (GWS) loci (pairwise *r*^2^ < 0.1) from the likelihood ratio test on the multinomial smoking trajectories (*p* < 5 × 10^−8^) (Supplementary Table 1) from the overall trajectory model. Seven of the 16 loci replicated previously reported associations with smoking or related phenotypes^10,11,23,24^. The most significant single nucleotide polymorphism (SNP) was rs7515828 (likelihood ratio test *p* = 6.29 × 10^−16^) near *LNC01360* on chromosome 1, which was recently reported to be significantly associated with smoking in a large GWAS from the UKBB and TAG^10^. Analysis for trajectory contrast I (current vs. never) showed minimal inflation (before correction *λ*_LDSC_ = 1.0281, se = 0.0096; after LDSC correction *λ*_LDSC_ = 0.9997, se = 0.0094; and after genomic control correction *λ*_LDSC_ = 0.8247, se = 0.0078). We identified 18 significant loci for trajectories of current vs. never with odds ratios (ORs) in the range of 0.90–1.09 (Table 2 and Supplementary Data 1) (Fig. 2 and Supplementary Fig. 1). The analysis for contrast II identified five significant loci for current vs. mixed with ORs ranged from 0.92 to 1.08 (before correction *λ*_LDSC_ = 1.0236, se = 0.0091; after LDSC correction *λ*_LDSC_ = 1.0000, se = 0.0089; and after genomic control correction *λ*_LDSC_ = 0.8868, se = 0.0081). Three genes–*CHRNA2, DBH*, and *DRD2*–were GWS in both contrast analyses (Fig. 2). Based on these findings, we believe that there is an association of *DRD2* with smoking trajectory, although previous findings on this relationship have been controversial^23,25–28^. These loci coincide with the most recent findings from large meta-GWAS^5,6^.

**Table 2:** Genome-wide significant associations for smoking trajectories in the Million Veteran Program (*N* = 286,118).

Smoking trajectory	POP	CHR:POS	SNP	EA	NEA	OR	*Z*	*P*
Contrast I (current vs. never)	EA	1: 28717871	rs61783804	T	C	0.94	−6.12	9E − 10
		1: 28717871	rs76509406	T	C	0.90	−5.65	2E − 08
		1: 73848331	rs7515828	C	T	0.93	−8.24	2E − 16
		2: 45159091	rs1004787	A	G	1.07	6.88	6E − 12
		2: 104496366	rs12477780	T	A	1.06	5.64	2E − 08
		2: 146118069	rs1474011	A	G	0.94	−6.75	1E − 11
		4: 152634121	rs28608075	C	T	0.92	−5.70	1E − 08
		6: 26391395	rs2237235	A	G	1.07	5.66	1E − 08
		6: 27021173	rs72838268	A	G	1.07	5.47	5E − 08
		7: 117593308	rs6969783	T	A	0.94	−6.65	3E − 11
		8: 27336767	rs2565060	A	T	1.09	7.13	1E − 12
		9: 136471660	rs112270518	G	A	0.92	−5.90	4E − 09
		11: 112826867	rs2212450	T	C	0.95	−6.08	1E − 09
		11: 113407114	rs3133388	G	A	1.07	6.95	4E − 12
		14: 33797853	rs11850899	C	T	0.94	−5.74	9E − 09
		15: 47844059	rs28505872	C	T	0.95	−5.78	7E − 09
		16: 69556715	rs889398	C	T	1.05	5.64	2E − 08
		16: 69953508	rs4985459	A	G	1.06	5.77	8E − 09
	AA	1: 4086827	rs4478781	C	T	1.11	5.72	1E − 08
	HA	6: 73193151	rs1334346	A	C	0.83	−5.76	8E − 09
Contrast II (current vs. mixed)	EA	5: 11232831	rs112030805	A	G	0.92	−5.60	2E − 08
		8: 27336978	rs2565059	G	A	1.08	7.14	9E − 13
		9: 136471660	rs112270518	G	A	0.93	−5.64	2E − 08
		11: 113344912	rs61902807	T	C	1.06	6.34	2E − 10
		19: 41339896	rs12459249	C	T	0.94	−7.68	2E − 14

*POP* ancestral population, *CHR* chromosome, *POS* position, *EA* effect allele, *NEA* noneffect allele, *OR* odds ratio, *Z* Wald test statistics, *P* Wald test *p* values, *EA* European American, *AA* African American, *HA* Hispanic American.

In the AA samples, we identified one locus for trajectory contrast I (current vs. never) with the lead SNP rs4478781 near to *LINC01346* (OR = 1.106, Wald test *p* = 1.06 × 10^−8^) (Table 2 and Supplementary Data 1) (Supplementary Fig. 2.1). A previous study reported that DNA hydroxymethylation of *LINC01346* was associated with a quantitative measure of neuritic plaque in Alzheimer disease in human postmortem brain^29^, suggesting a potential functional impact of this gene on brain pathology. No GWS variant was found for trajectory contrast II (current vs. mixed) in AAs.

In the HA samples, we identified one variant, rs1334346 near to *Regulating Synaptic Membrane Exocytosis 1* (*RIMS1*) that was significantly associated with smoking trajectory contrast I (OR = 0.831, Wald test *p* = 8.22 × 10^−9^) (Table 2 and Supplementary Data 1) (Supplementary Fig. 2.2). The protein encoded by *RIMS1* plays a role in the regulation of voltage-gated calcium channels during neurotransmitter release, and the variants in this gene were associated with autism^30^ and schizophrenia^31^. The analysis of contrast II (current vs. mixed) for smoking trajectory revealed no significant variant in HAs.

The trans-ethnic meta-GWAS combining EAs, AAs, and HAs in the MVP identified 14 GWS loci for contrast I (current vs. never) and 4 GWS loci for contrast II (current vs. mixed) (Supplementary Data 2) (Supplementary Fig. 3). The significant loci largely overlapped with the significant loci of the smoking trajectory analysis in EAs. For contrast I (current vs. never), 9 of 14 loci overlapped with the significant loci in EAs, and 5 loci were revealed only by smoking trajectory meta-analysis. For contrast II (current vs. mixed), the four loci identified by the trans-ethnic meta-GWAS were identical with the significant loci in EAs. Given that the sample sizes for AA and HA were relatively limited, subsequent analyses of smoking status and all downstream analyses focused on European-ancestry individuals.

### GWAS for smoking status

We first identified loci for smoking status in the EAs from the MVP sample using the phenotypes of smoking initiation (ever vs. never) and smoking cessation (current vs. past). Using a logistic regression model, a GWAS for smoking status showed very little inflation (*λ*_LDSC_ = 1.01 for initiation and *λ*_LDSC_ = 1.06 for cessation). Twelve loci were GWS for smoking initiation (Supplementary Data 1) (Supplementary Fig. 4), including three that were not seen in either the UKBB or GSCAN studies: *GRIK4, SPATS2, and FMNL3*. The GWAS for smoking cessation identified eight GWS loci (Supplementary Data 1) (Supplementary Fig. 4). Two of eight loci, rs34735365 near to *NR5A2* and rs77648866 near to *NICN1*, were associated only with smoking cessation and not with smoking initiation.

Of note, previous studies have shown significant associations in EAs of 15q25 (*CHRNA5-A3-B4*) and 8p11 (*CHRNB3-CHRNA6*) with CPD or FTND^32,33^. To validate these findings in the MVP sample, we conducted two GWASs for current CPD (*N* = 17,014) and past (*N* = 77,515) CPD phenotypes in EAs using MVP survey data, which yielded three GWS for each (Supplementary Table 2). The most significant signal for current CPD was a well-established SNP, rs12914385, located in an intron of *CHRNA3* on 15q25 (*β* = 0.014; Wald test *p* = 4.30 × 10^−10^). Another significant SNP, rs3025386, was near *DBH* (*β* = −0.017; Wald test *p* = 1.25 × 10^−8^), a gene previously associated with smoking cessation^3,24,34^. The third significant SNP—rs11697662 on chromosome 20—is a novel locus for current CPD (*β* = 0.016; *p* = 6.93 × 10^−9^). For past CPD, the most significant SNP, rs8040863, is a well-established smoking-associated exonic variant on *CHRNA3* (*β* = 0.044; Wald test *p* = 1.80 × 10^−20^). Rs12459249, proximal to *CYP2A6*, previously associated with the nicotine metabolite ratio^20^, was also GWS for past CPD (*β* = −0.033; Wald test *p* = 1.33 × 10^−11^). A novel locus for past CPD was rs7571606 on *TEX41* (*β* = 0.027; Wald test *p* = 4.62 × 10^−9^). Thus, our GWAS of CPD both replicated previously identified loci and yielded two novel loci for a quantitative smoking trait.

We performed meta-analyses on a combined sample size of 842,717 individuals from the MVP and GSCAN cohorts (excluding 23andMe) for two phenotypes separately: smoking initiation and smoking cessation. We identified 99 independent loci for smoking initiation and 13 loci for smoking cessation (Supplementary Data 3). For smoking initiation, our meta-analysis of MVP and GSCAN yielded 16 new loci, including rs10446671 near to *LOC101927285* and rs10211770 near to *LINC01441*. For smoking cessation, two of 13 loci were not reported in GSCAN: rs329120 near *JADE2* and rs12891477 near *LINC00637*.

### Downstream analyses and biological interpretation of GWAS results

As mentioned earlier, we applied downstream analyses to the association results of the pairwise trajectory contrasts and smoking status phenotypes obtained in EA samples in the MVP. Here we studied SNP-based heritability and heritability enrichments, gene prioritization and pathway enrichments for top signals identified in the GWASs, and summary statistics-based genetic correlation and potential causal–consequential relationships with other psychiatric and nonpsychiatric phenotypes.

Using LDSC^35^, the SNP heritability of smoking trajectory was 18.7% (SE = 0.010) for contrast I (current vs. never) and 5.8% (SE = 0.005) for contrast II (current vs. mixed). The heritability of smoking status in EAs was 6.9%, SE = 0.004 for smoking initiation and 6.1%, SE = 0.005 for smoking cessation (Supplementary Table 3). Thus, smoking trajectory I (current vs. never) accounted for more SNP heritability than smoking initiation while smoking trajectory II (current vs. mixed) has SNP heritability comparable to smoking cessation.

To estimate tissue and cell type-specific heritability, we conducted partitioned LD score regression using GenoSkyline-Plus functional annotations only in EAs^36,37^. LDSC showed significant heritability enrichments in multiple tissue and cell lines (Supplementary Data 4). In contrast I (current vs. never), the most significant heritability enrichment was for the anterior caudate in brain (enrichment score = 5.4, Wald test *p* = 4.7 × 10^−7^). Other significantly enriched cell types included the cingulate gyrus (enrichment score = 5.8, *p* = 1.5 × 10^−4^), normal human astrocytes (enrichment = 5.1, Wald test *p* = 1.1 × 10^−04^), and G-CSF-mobilized hematopoietic CD34+ stem cells (enrichment = 3.7, Wald test *p* = 9.0 × 10^−5^) (Supplementary Data 4). No significant heritability enrichment was identified for contrast II (current vs. mixed). The heritability of smoking status in the MVP-only sample showed significant enrichment in the anterior caudate for smoking initiation (enrichment = 5.7, Wald test *p* = 2.9 × 10^−8^) and in G-CSF-mobilized hematopoietic CD34+ stem cells for smoking cessation (enrichment = 5.3, Wald test *p* = 9.9 × 10^−5^) (Supplementary Data 4).

**Fig. 3:**
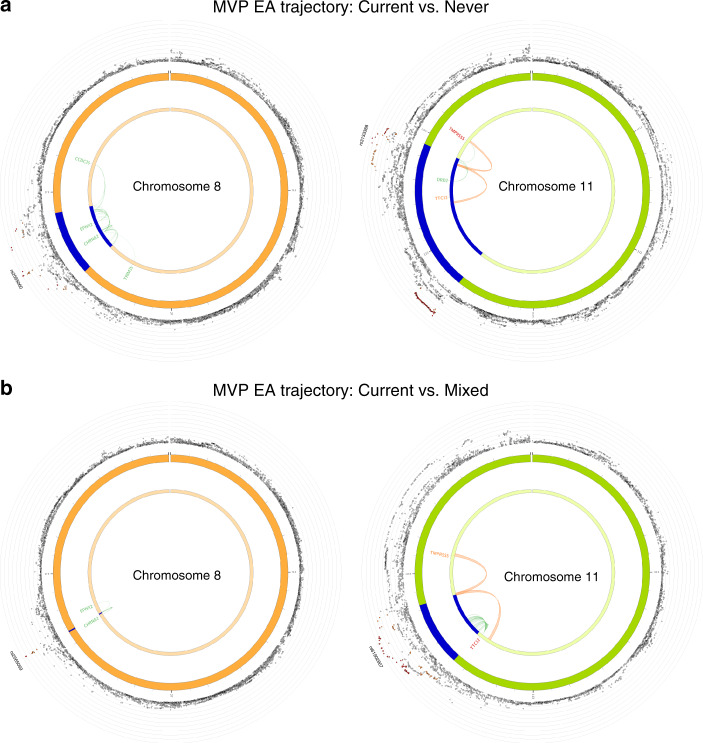
Gene prioritization for smoking trajectory contrasts. Functional mapping and annotation (FUMA) gene prioritization for smoking trajectory contrast I (current vs. never (**a**)) and contrast II (current vs. mixed (**b**)) in European American samples in the Million Veteran Program. The outer layer shows chromosomal Manhattan plots. Region in blue shows genome-wide significant locus. Genes mapped by chromatin interactions and eQTLs are colored in orange and green, respectively. Genes mapped by both chromatin interactions and eQTLs are colored in red.

To prioritize causal genes, we performed functional annotation of the GWAS-identified genetic variants for smoking trajectory in EA samples in the MVP by using co-localization analysis that focused on two molecular traits, expression quantitative trait loci (eQTLs) and histone interactions in brain tissues. We used the false discovery rate (FDR) < 0.05 for the eQTL mapping and FDR < 1 × 10^−6^ for the chromatin interaction mapping. Co-localization analysis was separately performed for smoking trajectories, contrast I (current vs. never) and contrast II (current vs. never) using FUMA GWAS. For contrast I (current vs. never), we identified 40 significant genomic regions co-localized among 18 significant loci and molecular traits (Supplementary Data 5). Positional mapping identified 16 significant genes, eQTLs were mapped to 28 genes in multiple brain regions including basal ganglia and hippocampus, and chromatin interactions were mapped to 21 genes in adult cortex. Importantly, co-localization analysis showed that several well-known genes for smoking, e.g., *DRD2, CHRNA2, TTC12*, were causal genes (Fig. 3). For contrast II (current vs. mixed), we identified five significant genomic regions. Positional mapping revealed three significant genes (*TTC12, CHRNA2*, and *CYP2A7*); eQTL mapping identified an additional significant gene, *EPHX2*, in the brain cerebellar hemisphere; and chromatin interaction mapping identified two significant genes, *TTC12* and *TMPRSS5* in adult cortex (Supplementary Data 5) (Fig. 3). The mapped brain cortex, hippocampus, and basal ganglia regions are functionally involved in reward reinforcement, cognitive processing, and emotion regulation and thus have implications for the etiology of addictive behaviors and their association with other neuropsychiatric disorders including depression^38–40^. These findings provide further support that GWAS for smoking trajectory identified biologically meaningful signals.

Using the Database for Annotation, Visualization and Integrated Discovery (DAVID)^41^ to conduct pathway analysis for smoking trajectories, we identified 23 significant pathways for smoking trajectory contrast I (current vs. never) and 4 for trajectory contrast II (current vs. mixed) (Supplementary Data 6). The top enriched pathways for trajectory contrast I (current vs. never) were identified for the histone *H1* gene cluster and were involved in DNA packaging complex, nucleosome assembly and organization, and chromatin assembly/disassembly activities. The top enriched pathways for trajectory contrast II (current vs. mixed) were identified for the *CYP2* gene cluster and were related to epoxygenase, oxidoreductase, and monooxygenase activities.

**Fig. 4:**
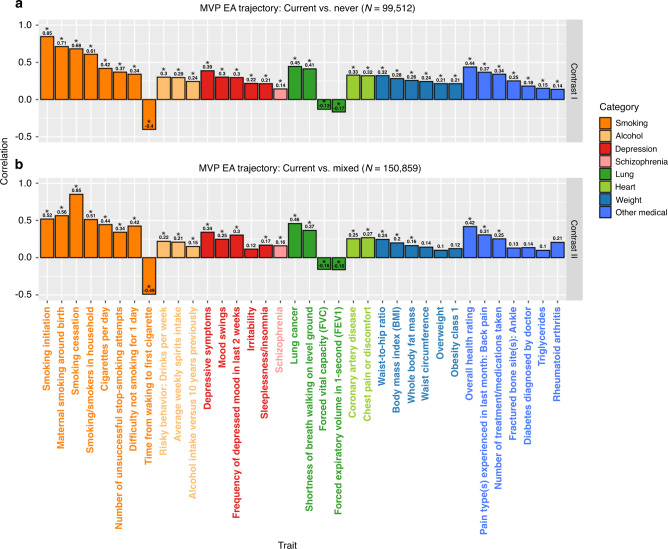
Genetic correlations between smoking trajectory contrasts and multiple complex traits. Complex traits with significant genetic correlations with smoking trajectory contrast I (current vs. never (**a**)) and contrast II (current vs. mixed (**b**)) in European American samples in the Million Veteran Program. Asterisk indicates significance after Bonferroni correction with a Wald test *p* value cutoff at 0.05/524/2 = 4.8 × 10^−5^. Detailed information on the exact *p* values, data source, and sample sizes for each complex trait is summarized in the Supplementary Data 7.

We used GeNetic cOVariance Analyzer (GNOVA) to estimate genetic correlations between smoking trajectory contrasts in EA samples from the MVP and 524 psychiatric and nonpsychiatric traits for which large-scale summary statistics are available^42,43^. We found 209 significant genetic correlations (Bonferroni-corrected *p* < 0.05/524/2 = 4.8 × 10^−5^) between at least one of the trajectory contrasts and other traits (Supplementary Data 7). As shown in Fig. 4, smoking trajectory contrasts were significantly genetically correlated with other smoking-related phenotypes, alcohol phenotypes, and psychiatric disorders, including positive correlations with depressive symptoms and schizophrenia. There were also significant positive genetic correlations between smoking trajectory contrasts and lung disease, coronary artery disease, diabetes, obesity, and negative correlations between smoking and overall health rating. All of these findings are expected and consistent with previous reports.

To understand causal–consequential relationships between smoking trajectory and significantly genetically correlated traits, we performed a bi-directional two-sample MR analysis for smoking trajectory contrast I (current vs. never) and contrast II (current vs. mixed) separately. Here, significant SNPs from the trajectory GWAS served as an instrumental variable, smoking trajectory as an exposure, and a psychiatric or nonpsychiatric trait as an outcome variable; MR analyses were also performed with smoking trajectory as an outcome and the various complex traits as exposures. Trait pairs with three or fewer instrumental variables were excluded from the analysis. We used four MR methods: inverse-variance weighted (IVW), weighted median (WM), MR-Egger regression (MR-Egger), and MR-PRESSO to test the causal relationships. We applied a Bonferroni correction to correct for different MR methods and all combinations of causal paths. We present causal paths for statistically significant traits identified by one of the four MR methods.

We identified five significant causal paths using trajectory contrast I (current vs. never) as an exposure and 23 significant causal paths using it as an outcome. While no significant results identified using trajectory contrast II (current vs. mixed) as an exposure, 12 were identified as being significant using it as an outcome (Supplementary Data 8). MR-Egger seemed to be the most conservative and identified no significant causal relationship or pleiotropic effect. While the significance varied across methods, the magnitude and direction of estimated causal effects were highly consistent across IVW, median weighted, and MR-PRESSO. Our analyses suggested multiple interesting causal–consequential relationships. For example, in trajectory contrast I (current vs. never), smoking was a cause of wheeze or whistling in the chest in last year, while depression (fed-up feelings) likely led to smoking. Smoking was an outcome of multiple traits related to body weight including body mass index and whole-body fat mass. Interestingly, we found that higher education likely had a causal effect on smoking behavior. We found no trait pair with both significant forward and reverse causal relationships. It is noted that the statistical significance is inadequate to claim a causal relationship between trait pairs. The identified significant MR results serve as suggestive evidence for further investigation and the causal relationships should be interpreted with caution.

## Discussion

We present data from a GWAS of smoking phenotypes in the MVP, the largest relatively homogeneous clinical sample studied to date, and combine these results with a published study to yield a large meta-analysis for smoking status. We identified more than a dozen significant genetic risk loci for smoking trajectories in the MVP. A meta-GWAS of MVP and GSCAN in European-ancestry individuals identified more than 100 loci for smoking status, including loci that were not seen in either MVP or GSCAN alone. The loci identified in the MVP cohort account for more genetic heritability in smoking behavior than previous studies^5,10^. The heritability was enriched in brain regions related to smoking behavior. We prioritized 40 genes strongly associated with biological functions relevant to smoking trajectory of current vs. never, including neural and synaptic function. Smoking-associated loci were highly correlated with 209 smoking-related psychiatric and nonpsychiatric phenotypes. We found 33 significant causal relationships between smoking trajectory and other smoking phenotypes, alcohol consumption, and psychiatric traits. Together, our results provide new insights into genetic risks for smoking behavior and implicate loci to be validated biologically in future studies.

We leveraged robust EMR data to yield a longitudinal phenotype for the trait of smoking trajectories. This novel phenotype identified genomic loci for smoking trajectories overlapping with those from a meta-analysis of smoking status in a much larger sample^5,10^. Thus, longitudinal smoking trajectories may offer greater power to detect novel genetic variants than the widely used binary phenotype of ever vs. never smoked. We identified several interesting loci for smoking trajectory. A locus containing neuronal growth regulator 1 (*NEGR1*) was associated with the smoking trajectory of current vs. never. *NEGR1* encodes a protein involved in neuronal development and maturation^44,45^; the gene has been associated with major depression^46^ and showed altered gene expression in the prefrontal cortex of schizophrenic patients^47^. A variant in *NEGR1* was also previously linked to obesity^48^, which, like smoking, involves a dysregulation of appetitive behavior. Several loci associated to smoking trajectory were also associated with other relevant phenotypes. A locus containing *CNNM2*, which was significantly associated with smoking trajectories in this study, was recently linked to smoking behavior in a large meta-analysis^6^. A variant in *CAMKMT* for smoking trajectories in the MVP was previously associated with anxiety disorder^49^.

Some of the genes identified in the meta-analysis for smoking status are particularly noteworthy. For example, rs6265 on *BDNF* (Wald test *p* = 1.3 × 10^−14^) is a well-established exonic functional SNP for psychiatric disorders^50,51^, including smoking behavior. One of the loci located in *NRXN1* (*Neurexin 1*, index SNP rs11125335, Wald test *p* = 1.1 × 10^−11^) was previously associated to nicotine dependence^52^. Neurexins are cell-surface receptors that bind neuroligins to form Ca(2+)-dependent neurexin/neuroligin complexes at synapses in the central nervous system^53^. This locus was previously identified as a candidate gene but was not GWS in the TAG samples^12^. Genetic variation in *NRXN1* has also been linked to autism spectrum disorder^54^, attention deficit hyperactivity disorder^55^, and schizophrenia^56^. One of the significant signals for smoking status was from *NCAM1*, index SNP rs7110863 (Wald test *p* = 1.6 × 10^−35^), which was highly significant for smoking trajectory in the MVP (index SNP rs7126748; Wald test *p* = 4.2 × 10^−8^). *NCAM1* is located in a well-known genomic region on chromosome 11, *NCAM1-TTC12-ANKK1-DRD2*, previously associated with nicotine dependence^28^ and smoking motives^57^.

Of note, despite several recently reported large GWASs for smoking traits^5,10^ that revealed more loci than the present study, the estimated heritability of smoking for trajectory contrast I (current vs. never) in our study is substantially higher than that in previous studies (>15% for contrast I vs. <10% for other smoking traits). These results suggest that smoking trajectories are a more powerful trait for detecting genome-wide signals with small effects on smoking behavior. A higher SNP heritability level in our sample also suggests that meta-GWAS from the multiple cohorts may be missing vulnerability for smoking traits, possibly due to heterogeneity in the meta-GWAS. Note that our results estimated with LDSC were based on a heritability model that assumes that all genetic variations contribute equally to the SNP-based heritability and the summary statistics were derived after adjusting for age, sex, and top ten PCs as covariates. The heritability estimation is subject to inadequate covariate adjustment and novel methods developed to provide a more flexible specification of heritability model are worth future investigation^58–60^.

We were unable to replicate the identified loci associated with smoking trajectories in an independent sample due to a lack of a replication cohort with comparable longitudinal measurements of smoking behavior. We identified only one locus for smoking trajectory in the AA and HA populations, likely due to the modest sample sizes in these populations. The findings from trans-ancestry meta-analysis mostly reflect the findings from the EA samples. In addition, the majority of our samples were men, so that it was not feasible to examine sex-specific genetic risks.

In conclusion, compared to more widely used, simple smoking phenotypes, we demonstrated that (1) longitudinal EMR data permit the estimation of smoking trajectories, and (2) these phenotypes yield greater statistical power to detect small-effect variants, enabling the detection of novel genetic risk variants for smoking behavior.

## Methods

### Study cohort

The MVP recruited veteran volunteers and collects data from questionnaires, EMRs, and blood samples for genomic analysis. The Central Veterans Affairs Institutional Review Board (IRB) and site-specific IRBs approved the MVP study. All relevant ethical regulations for work with human subjects were followed in the conduct of the study and informed consent was obtained from all participants.

We selected 26,497 LD pruned SNPs and applied flashpca version 1.2.5 to perform PC analysis on 343,268 unrelated MVP samples and 2504 1000 Genomes Project (1KG) samples to identify population structure. The annotated Euclidean center of European, African, admixed American, East Asian, and South Asian 1KG samples plus the admixture analysis results with a probability cutoff of 0.8 were used to define populations. We removed samples with a high genotype missing rate (>10%), discordant sex, excessive heterozygosity (>3 sd), and up to second-degree relatives. A total of 209,915 EAs, 54,867 AAs, and 21,336 HAs passed quality control filters. The mean age of the study cohort ranged from 58 to 64 years across three ancestral groups (EA: mean = 64, standard deviation (SD) = 13, AA: mean = 58, SD = 12, and HA: mean = 56, SD = 15) and majority of the MVP samples were male (EA: 93%, AA: 87%, and HA: 92%).

### Smoking phenotypes

Smoking data from 2000 to 2015 were obtained from the Veteran Healthcare Administration Corporate Data Warehouse. Details on the data extraction methods are provided elsewhere^61^. In brief, EMR smoking data are collected nationally from patients approximately annually using the clinical reminder process, which prompts providers to ask patients questions related to health. EMR smoking data consist of text values that represent responses to the specific smoking-related queries to patients, which can vary by site and over time. Mapping strategies were created to classify these responses into never, past, and current smoking status and can be found on www.vacohort.org^61^.

Smoking trajectory phenotype accounts for variation in smoking status over time^22^. We used joint trajectory modeling to sort each participant’s smoking values (current, past, never) into clusters and estimated distinct trajectories^62–64^. We used age as the time scale to account for possible decreases in smoking with age. The procedure calculated each individual’s probability of belonging to each trajectory and assigned the individual to the trajectory with the highest probability of membership (mostly current smoking, mixed smoking and nonsmoking, mostly never smoking). Applying the phenotype definition, we identified 40,456 mostly current smokers, 110,403 mixed smokers, and 59,056 mostly never smokers in EAs, 13,511 mostly current smokers, 23,605 mixed smokers, and 17,751 mostly never smokers in AAs, and 2920 mostly current smokers, 11,221 mixed smokers, and 7195 mostly never smokers in HAs.

Using all available EMR smoking observations, we used the most common (modal) value for smoking status assessment. We identified 72,729 never smokers, 71,002 past smokers, and 66,184 current smokers in the EA sample. We further contrasted ever smoked (past or current smokers) with never smoked (nonsmokers) to study smoking initiation behavior. Similarly, we used the modal value of smoking status classification and contrasted current with past to reflect smoking cessation phenotype.

Cigarettes per day was based on responses to the MVP baseline survey questionnaire. The subjects were asked, “Do you currently smoke cigarettes?”, If yes, then “How many cigarettes do you smoke per day now?”. If no, then “Over the entire time you smoked, on average, how many cigarettes did you smoke per day?”. The responses were on a scale from 1 to 5, corresponding to (1) less than a half pack, (2) a half pack, (3) 1 pack, (4) 2 packs, and (5) more than 2 packs. We identified 17,014 and 77,515 individuals for CPD current and CPD past with nonmissing responses, respectively.

### Genotyping, imputation, and quality control

MVP used an Affymetrix Axiom Biobank Array that genotyped ~723,000 markers, which were enriched for exonic variants. SNPs were validated for common diseases and phenotypes of specific interest to the VA population (e.g., psychiatric traits)^65^. Minimac3 and the 1000 Genomes Project 3 reference panel were used to conduct genotype imputation, which resulted in ~79 million variants in this study^66^. We filtered out rare variants (minor allele frequency < 0.01), variants with a missing rate > 5%, variants with imputation *r*^2^  < 0.8, and those that deviated significantly from Hardy–Weinberg equilibrium (*p* < 1 × 10^−6^).

### Single nucleotide polymorphism (SNP) association analysis

For the trajectory GWAS, we applied a multinomial regression analysis using SNPTEST (v2.5.4-beta2) to test for an association of SNPs with trajectories. The overall fit of the model was evaluated using the likelihood ratio test, which assessed the strength of the relationship between each genetic variation and the multinomial smoking trajectory groups by comparing the fit of two models with and without the genetic variation. The two trajectory contrasts were modeled simultaneously, which resulted in smaller standard error estimates of genetic effects and thus greater statistical power. We estimated genetic effects for two trajectory contrasts: contrast I (current vs. never) and contrast II (current vs. mixed). For the smoking status phenotype modal value that defined smoking initiation and smoking cessation, we applied logistic regression to estimate marginal effects of each single genetic variant on smoking. For the CPD phenotype, we used linear regression analysis. We used PLINK (v1.9) to performed logistic and linear regression association analyses. In each model, we included age and sex as covariates and the ten top PCs calculated with flashpca (v1.2.5) to adjust for population stratification. Quantile–quantile plots were generate to evaluate the extent to which the observed GWAS *p* values deviated from the null hypothesis (Supplementary Fig. 5). The LD score regression intercept was used to quantify inflation resulting from confounding bias^35^. We identified proxy independent SNPs (LD, *r*^2^ < 0.1) and selected the ones with the most significant *p* values as index SNPs. We then defined a risk locus as a physical region containing all GWS variants (*p* < 5 × 10^−8^) that were in LD (*r*^2^ > 0.6) with the index SNP. Loci within 250 kilobases were merged^67,68^. We used ANNOVAR (v2016Feb01) to annotate index SNPs with their nearest genes^69^. Loci mapped to the same nearest gene were merged to yield one risk locus. We summarized whether the identified locus in the MVP was mapped to a gene previously reported to be associated with smoking phenotypes in other studies. If it was, we also listed the associated smoking phenotypes. The results are based on a literature review and query search in the GWAS Catalog^70^. LocusZoom (v1.3) was used to visualize regional associations and LD patterns^71^.

### Meta-analysis of GWAS results

We meta-analyzed GWAS summary statistics of smoking status phenotypes (smoking initiation meta *N* = 842,717, smoking cessation meta *N* = 450,129) in the MVP EA samples (*N* = 209,915), and GSCAN excluding 23andMe samples (*N* = 632,802). The summary statistics from 23andMe are not publicly available. We also performed trans-ethnic meta-analyses of the trajectory phenotypes (contrast I meta *N* = 140,889, contrast II meta *N* = 202,116) in EAs, AAs, and HAs in the MVP. Meta-analyses were performed using inverse-variance mixed effects model implemented in METAL (v2011-03-25) with the test statistics and standard errors scheme^72^. We enabled the genomic control correction option on all GWAS results in the meta-analysis.

### Downstream analysis for smoking GWASs in European Americans

Heritability estimation and enrichment analyses were performed for smoking initiation, smoking cessation, smoking trajectory contrast I (current vs. never), and smoking trajectory contrast II (current vs. mixed) in the MVP EA samples. We applied LD score regression (v1.0.0) to estimate the narrow-sense heritability due to additive genetic effects^35^. We also estimated heritability enrichments to identify tissue and cell types that were the most relevant to smoking-related traits^36^. We used 66 functional annotations from GenoSkyline-Plus (v1.0.0) including tissues and cell lines from blood, brain, lung, vascular, heart, thymus, spleen, muscle, gastrointestinal, pancreas, liver, fat, bone/connective, skin, breast, and ovary for the heritability enrichment analysis^37^. Bonferroni correction was applied to the 66 enrichment tests for two smoking trajectory contrasts and two smoking status phenotypes, resulting in a significance cutoff of 0.05/66/4 = 1.9 × 10^−4^.

We performed functional gene mapping for two smoking trajectory contrasts in the MVP EA samples. Positional, eQTL, and chromatin interaction mapping was performed using the functional mapping and annotation tool FUMA (v1.3.6)^73^. We used the default parameter settings for the identification of lead and candidate SNPs except for changing the maximum distance to 250 kilobases to merge LD blocks into a locus. The change was consistent with the distance parameter we used in previous genetic loci definition. Positional mapping was performed with the default parameter setting. eQTL mapping was restricted to 13 GTEx v8 brain tissues. We used the built-in adult cortex Hi–C data and enhancer/promoter annotations in 12 brain tissues from Roadmap epigenomes to perform chromatin interaction mapping. By default, we used FDR < 0.05 for the eQTL mapping and FDR < 1 × 10^−6^ for the chromatin interaction mapping. The functional annotation and gene mapping parameter setting and results are available on the FUMA public results section.

We used DAVID (v6.8) to perform pathway-enrichment analysis on GWAS variants with *p* values less than 1 × 10^−5^^41^. ANNOVAR was used to map variants to their nearest genes^69^. Smoking trajectory contrast I (current vs. never) had 344 genes mapped from 6296 variants and smoking trajectory contrast II (current vs. mixed) had 130 genes mapped from 877 variants. Gene Ontology (GO) terms in three categories (GO: biological process, GO: cellular component, and GO: molecular function) were used for pathway-enrichment analyses and biological interpretation^69^. From 550 pathway-enrichment tests, 321 and 229 GO terms contained genes that overlapped with smoking trajectory contrast I (current vs. never) and smoking trajectory contrast II (current vs. mixed), respectively. Bonferroni correction was applied to correct for 550 tests, yielding a significance cutoff of 0.05/550 = 9.1 × 10^−5^.

We used GNOVA to estimate genetic correlations between smoking trajectory phenotypes and other traits and diseases with publicly available summary statistics^42^. GWAS summary statistics for 524 complex traits and diseases were downloaded from LD Hub and recent publications on addictive behaviors^43^. For the UKBB phenotypes integrated in LD Hub, we manually filtered out lifestyle and environmental traits with a keyword search for sport, activity, exercise, driving, transportation, travel, job, and employment. Bonferroni correction was applied to the 524 correlations and two smoking trajectory contrasts, yielding a significance cutoff of 0.05/524/2 = 4.8 × 10^−5^.

Bi-directional Mendelian randomization (MR) was performed on trait pairs identified with significant genetic correlations. Instrumental variables were selected as GWS SNPs for the exposure excluding those GWS for the outcome to satisfy the MR assumptions. Trait pairs with 3 or fewer instrumental variables were excluded from the analysis, leaving 638 trait pairs to be tested for causal relationships. We used four MR methods: IVW, WM, MR-Egger, and MR-PRESSO implemented in the MendelianRandomization (v0.4.2) and MR-PRESSO (v1.0) R packages^74–77^. We tested the intercept term from the MR-Egger to detect potential horizontal pleiotropic effects. We also used MR-PRESSO to identify outliers of instrumental variables with pleiotropic effects. The significance cutoff for outlier identification was calculated as 0.05/number of instrumental variables. If outliers were identified, we reported causal estimates obtained by MR-PRESSO after removing the outliers. In the absence of outliers, the MR-PRESSO provided results identical to those from IVW. We used *t*-test statistics to derive *p* values from all four methods for comparability. We applied a Bonferroni correction on bi-directional MR tests on trait pairs with more than three instrumental variables and each was tested with four methods, resulting in a significance cutoff of 0.05/638/4 = 1.96 × 10^−5^.

### Reporting summary

Further information on research design is available in the Nature Research Reporting Summary linked to this article.

## Supplementary information

Supplementary Information

Descriptions of Additional Supplementary Files

Supplementary Data 1

Supplementary Data 2

Supplementary Data 3

Supplementary Data 4

Supplementary Data 5

Supplementary Data 6

Supplementary Data 7

Supplementary Data 8

Reporting Summary

## Data Availability

The full summary-level association data from the meta-analysis for each of the smoking-related traits from this report are available through dbGaP accession number phs001672.v4.p1. 1000 Genomes Project reference panel can be downloaded from [ftp://ftp.1000genomes.ebi.ac.uk/vol1/ftp/]. GWAS summary statistics used in the genetic correlation analysis were made publicly available by GSCAN, Social Science Genetic Association Consortium (SSGAC) [https://www.thessgac.org/data], and LD Hub.

## Code availability

Code to perform analyses in this paper is available from the authors upon request (B.L.), or from the URLs associated with each software in the online methods section.
