# Supplementary material for: Genome-wide association study of smoking trajectory and meta-analysis of smoking status in 842,000 individuals
Source: Nat Commun. 2020 Oct 20;11:5302. doi: 10.1038/s41467-020-18489-3 (PMC7598939; doi:10.1038/s41467-020-18489-3)
Supplement: Supplementary file 1 — Supplementary Information [file 41467_2020_18489_MOESM1_ESM.pdf]

Genome-wide association study of smoking trajectory phenotypes and meta-analysis of smoking status in 842,000 Individuals

Xu, Li et al.

Supplementary Figure 1.1

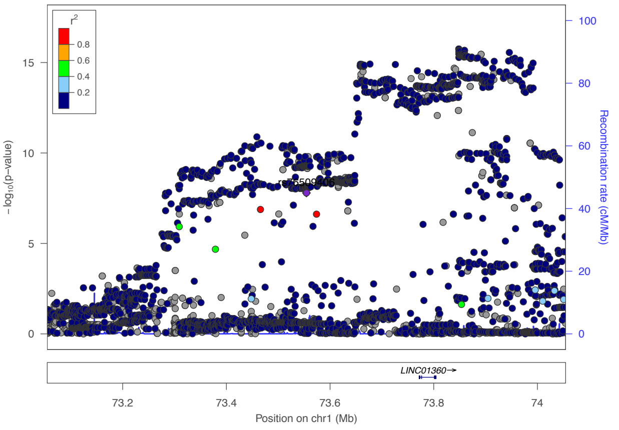

Supplementary Figure 1.2

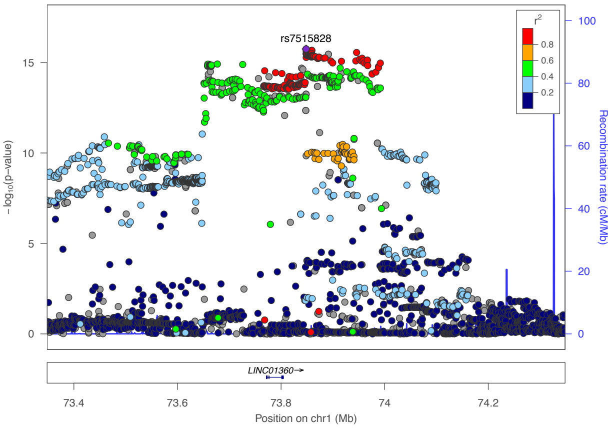

Supplementary Figure 1.3

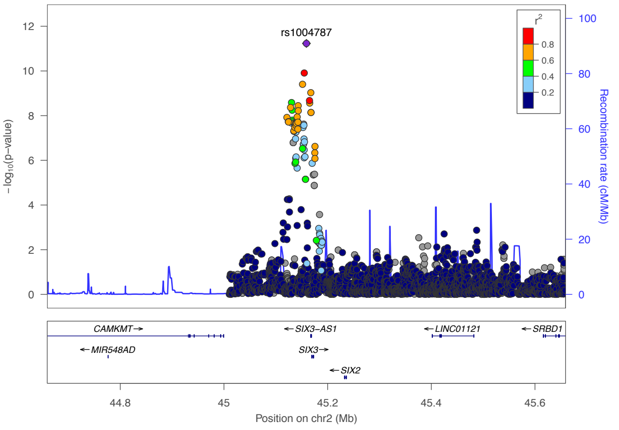

Supplementary Figure 1.4

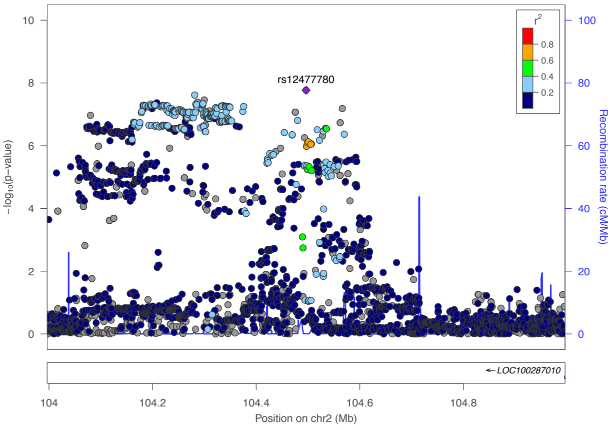

Supplementary Figure 1.5

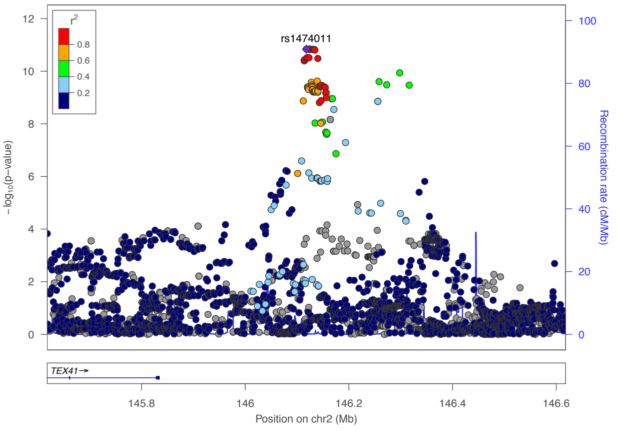

Supplementary Figure 1.6

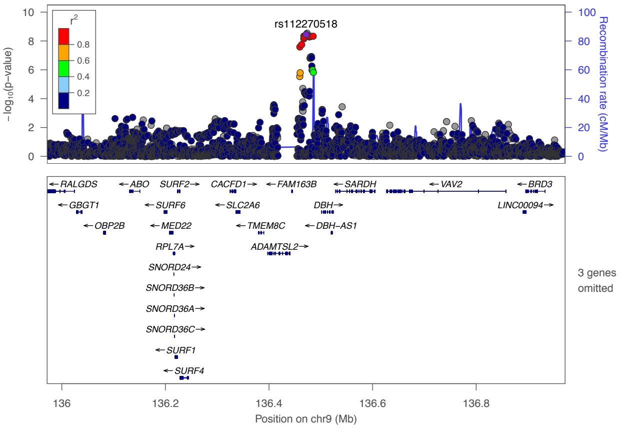

Supplementary Figure 1.7

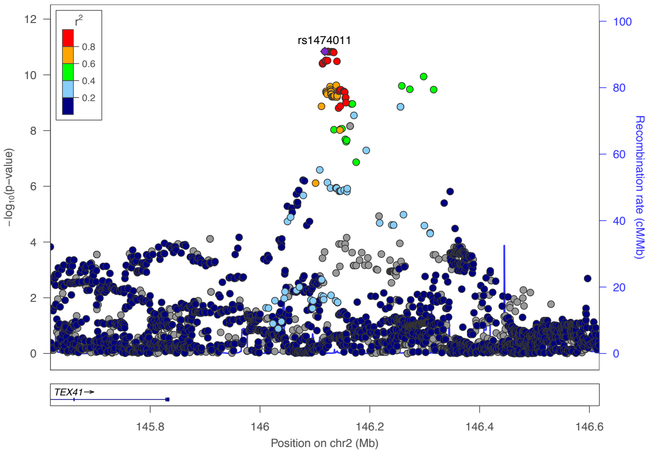

Supplementary Figure 1.8

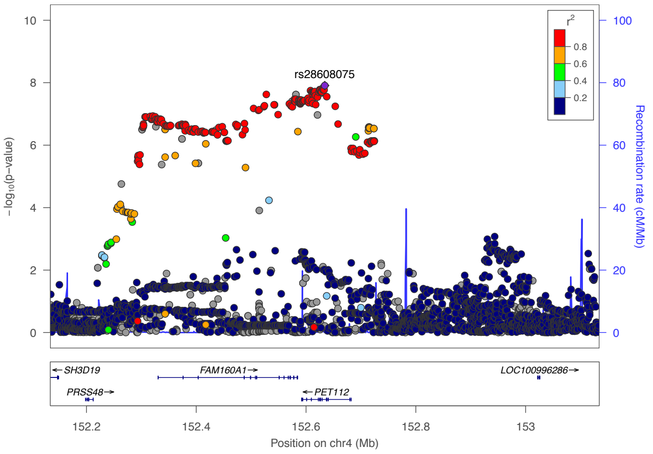

Supplementary Figure 1.9

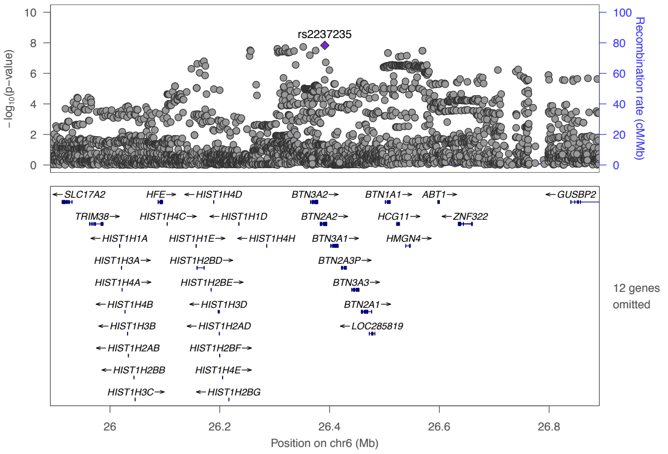

Supplementary Figure 1.10

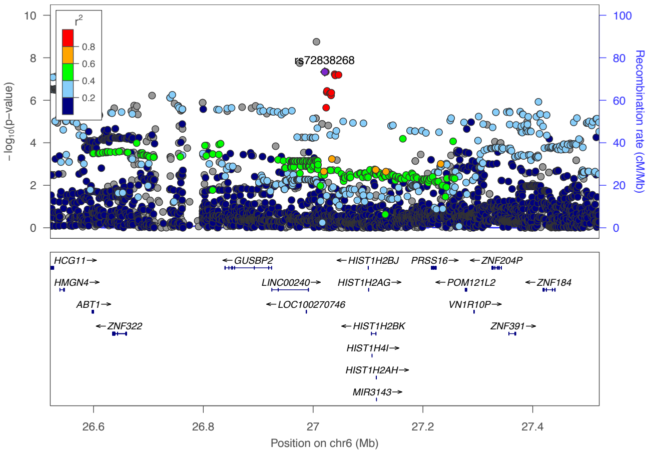

Supplementary Figure 1.11

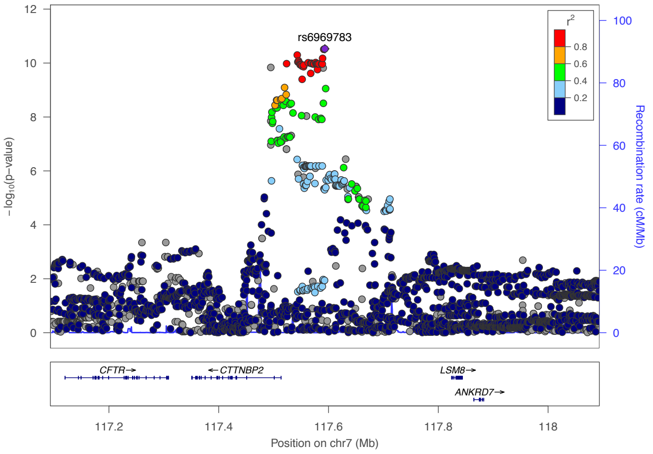

Supplementary Figure 1.12

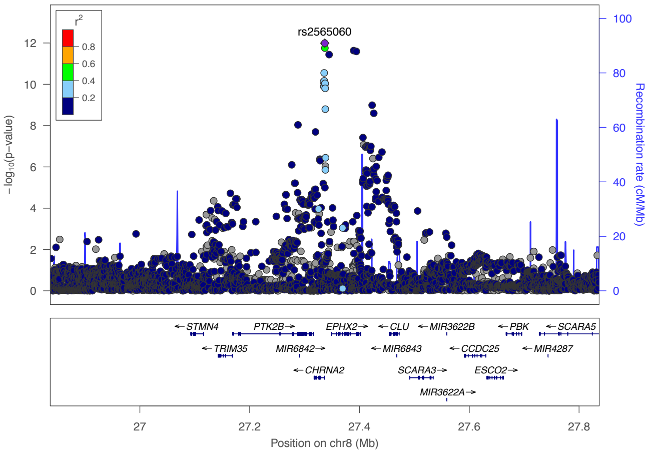

Supplementary Figure 1.13

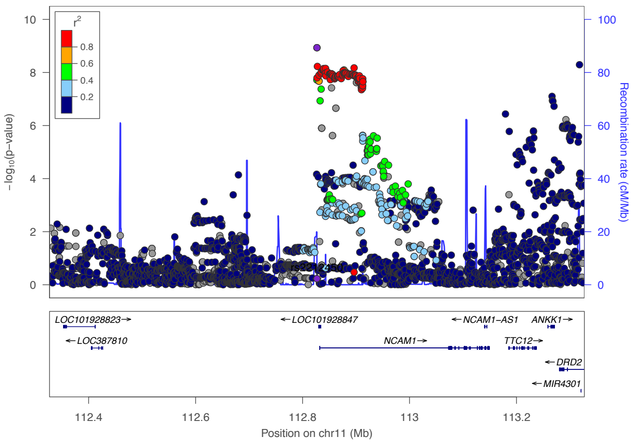

Supplementary Figure 1.14

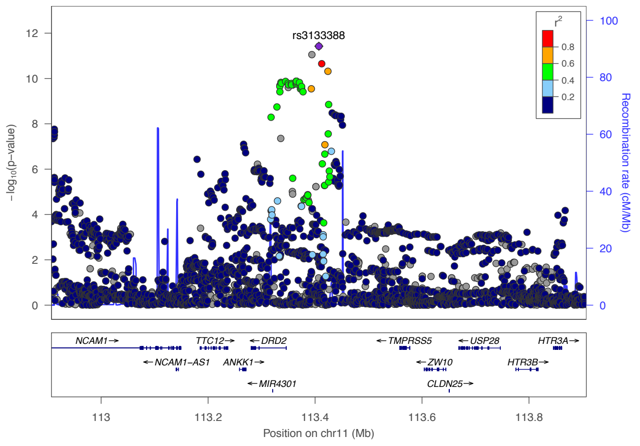

Supplementary Figure 1.15

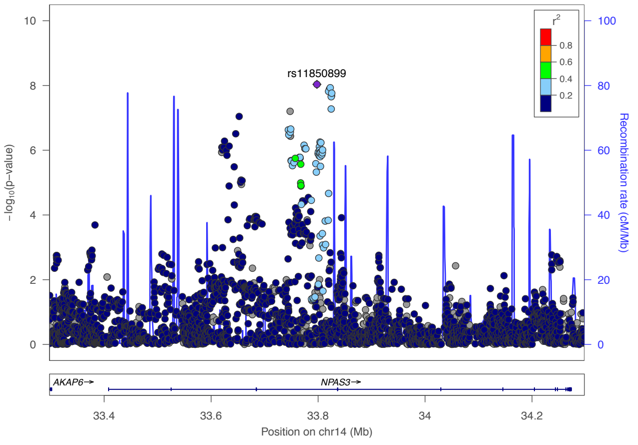

Supplementary Figure 1.16

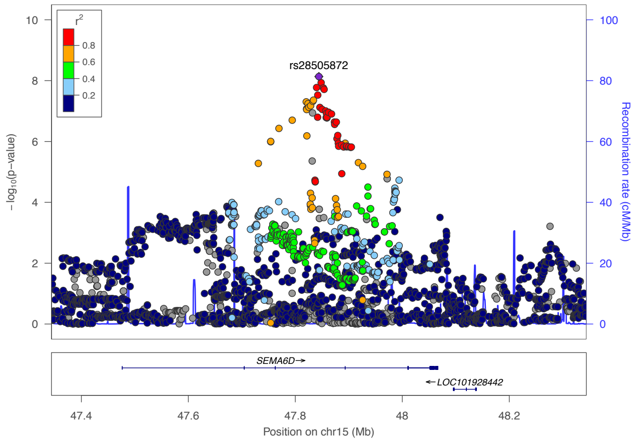

Supplementary Figure 1.17

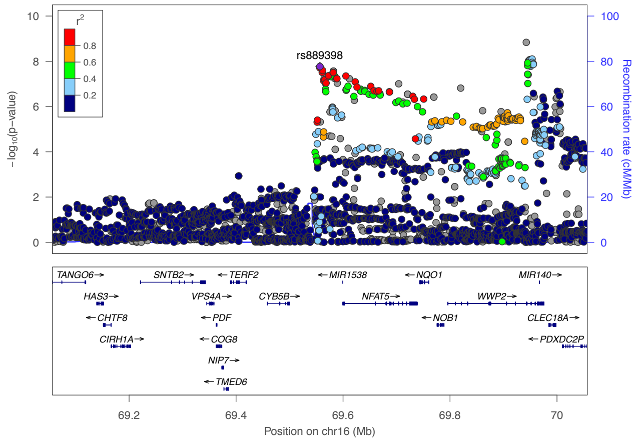

Supplementary Figure 1.18

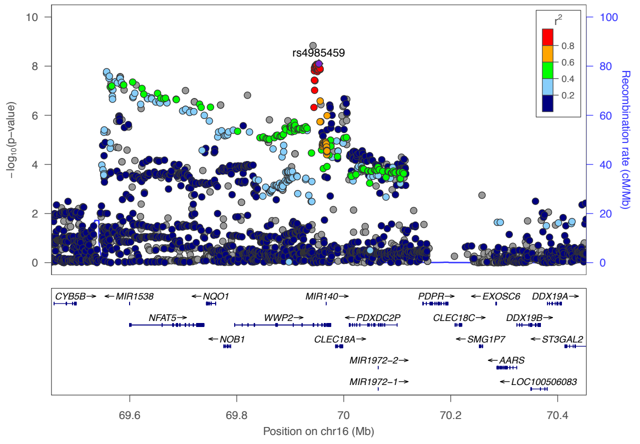

Supplementary Figure 1.19

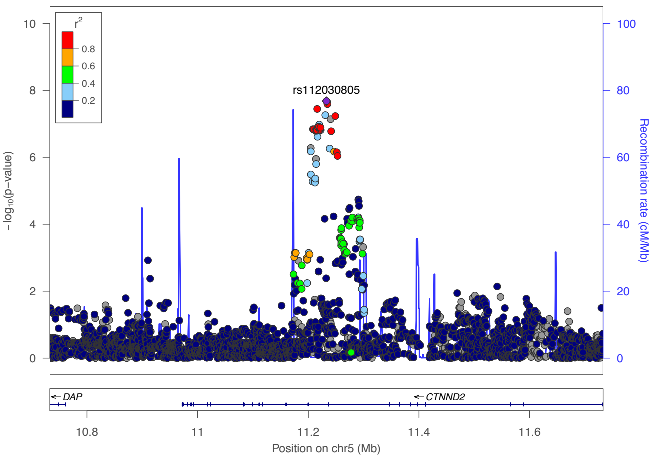

Supplementary Figure 1.20

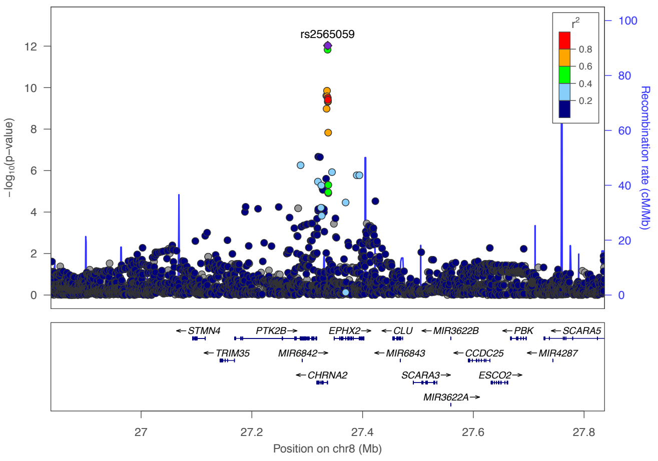

Supplementary Figure 1.21

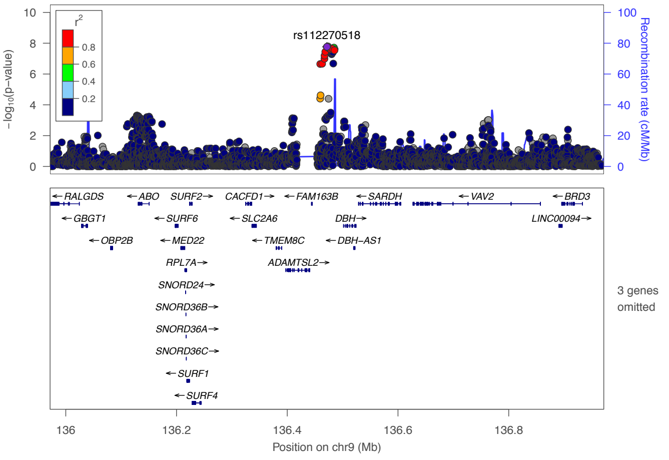

Supplementary Figure 1.22

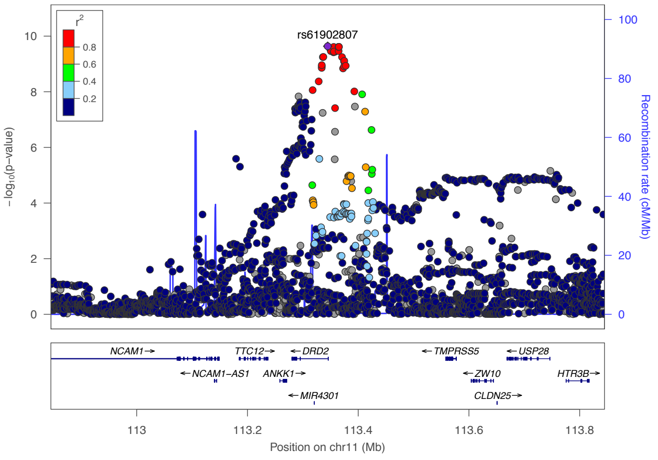

Supplementary Figure 1.23

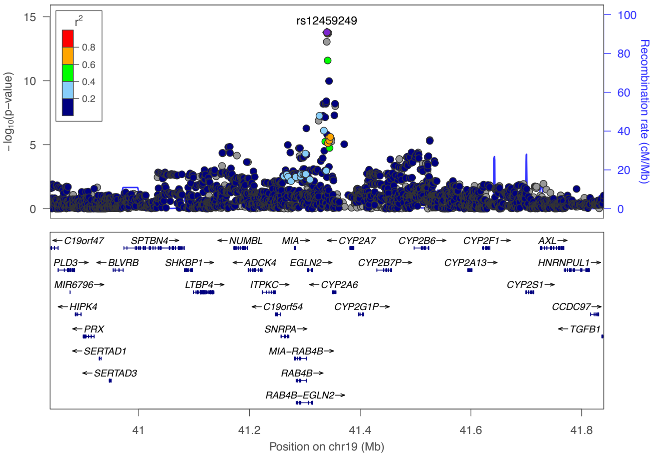

Supplementary Figure 1. LocusZoom plots for the genome-wide significant loci identified for smoking trajectory contrasts in European American samples in the Million Veteran Program. Smoking trajectory contrast I (current vs. never, Supplementary Figure 1.1–1.18) and contrast II (current vs. mixed, Supplementary Figure 1.19–1.23). Loci were arranged in the same order as in Table 2.

Supplementary Figure 2.1

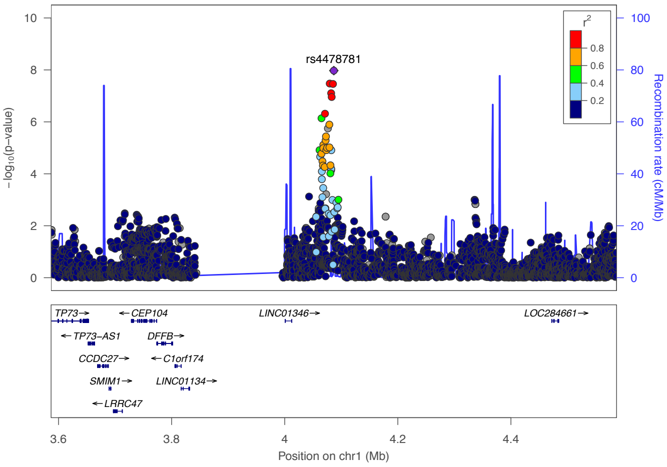

Supplementary Figure 2.2

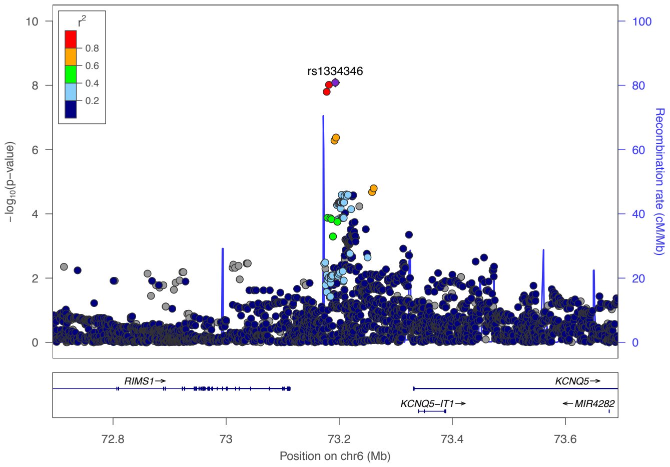

Supplementary Figure 2. LocusZoom plots for the genome-wide significant loci identified for smoking trajectory contrasts in African American and Hispanic American samples in the Million Veteran Program. Smoking trajectory contrast I (current vs. never) in African Americans (Supplementary Figure 2.1) and Hispanic Americans (Supplementary Figure 2.2).

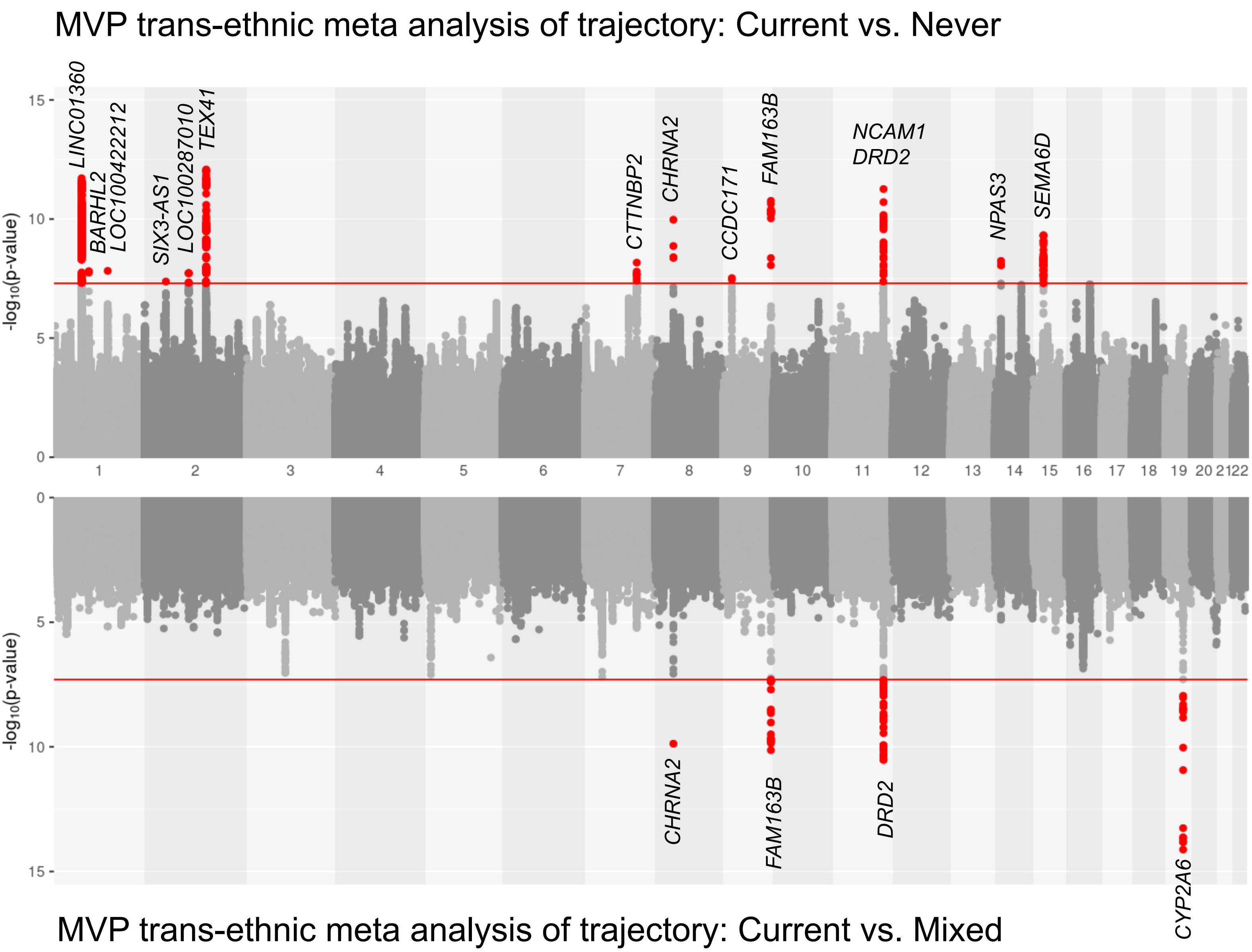

Supplementary Figure 3. Mirror Manhattan plots for smoking trajectory contrasts in the trans-ethnic meta analysis of European American, African American, and Hispanic American samples in the Million Veteran Program. Smoking trajectory contrast I (current vs. never, upper) and contrast II (current vs. mixed, lower). Genome-wide significant loci were highlighted in red and mapped to genes by nearest location.

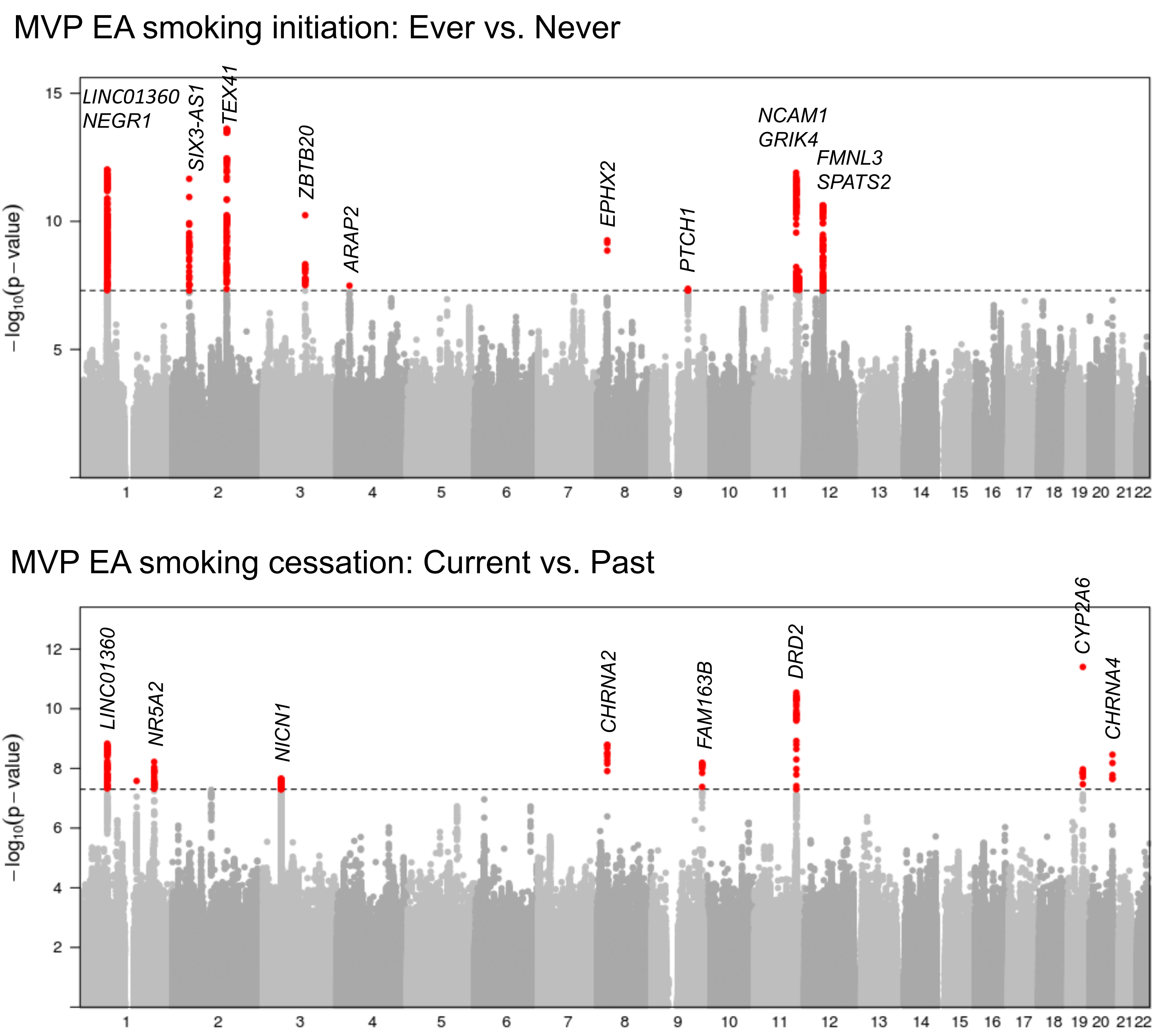

Supplementary Figure 4. Manhattan plots for smoking status phenotypes in European American samples in the Million Veteran Program. Smoking initiation (ever vs. never, upper) and smoking cessation (current vs. past, lower). Genome-wide significant loci were highlighted in red and mapped to genes by nearest location.

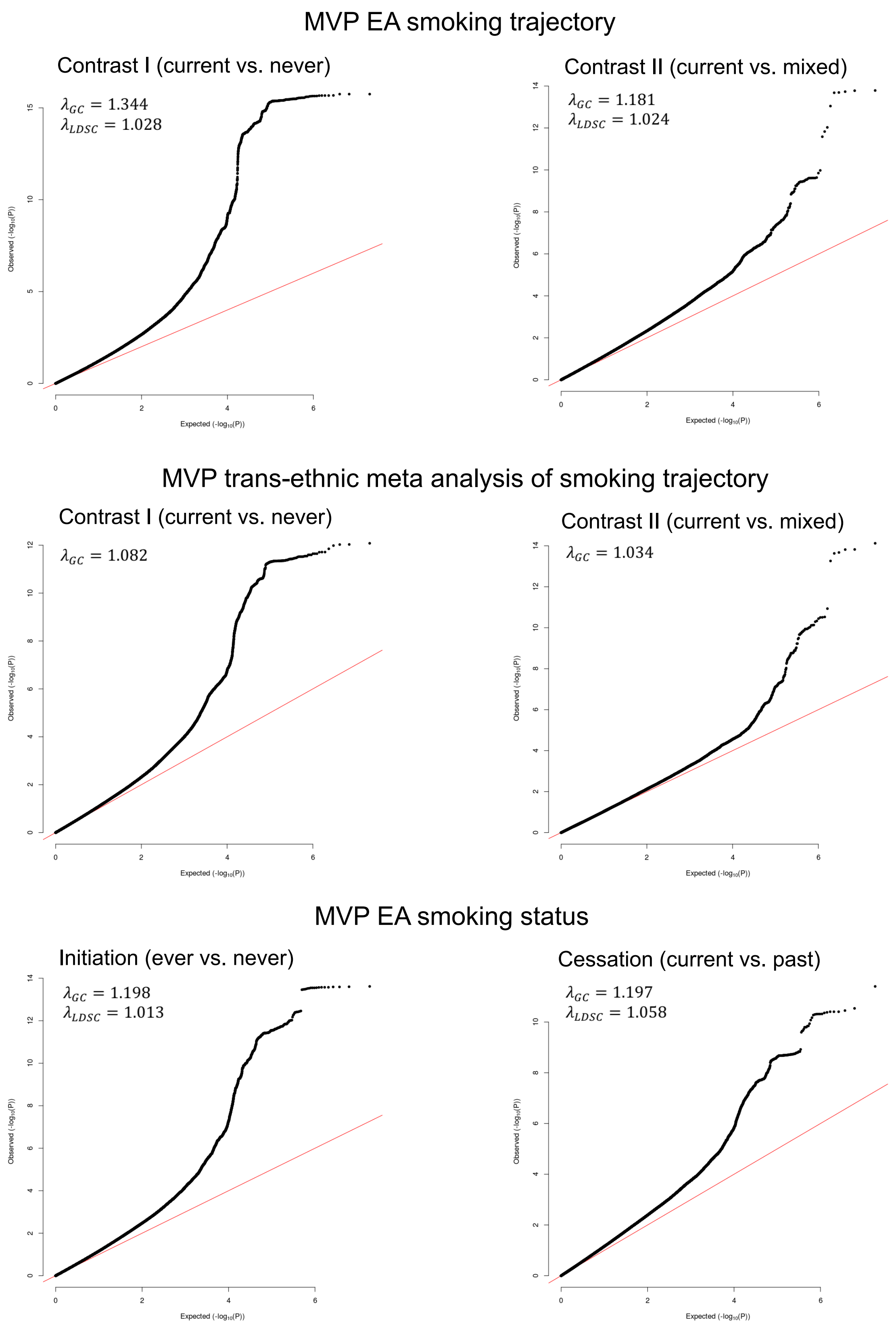

Supplementary Figure 5. Quantile-quantile (QQ) plots for the genome-wide association studies (GWAS) of smoking phenotypes in the Million Veteran Program. Smoking trajectory contrasts in European American samples (upper), trans-ethnic meta analysis of smoking trajectory contrasts in European American, African American, and Hispanic American samples (middle), and smoking status in European American samples (lower). Genomic inflation factor λ_{GC} and/or intercept from the LD score regression λ_{LDSC} were calculated to measure inflation of GWAS results.

Supplementary Table 1. Genome-wide significant associations for multinomial smoking trajectories (likelihood ratio test) in European Americans in the Million Veteran Program

Chromosome	SNP	Position	Reference_allele	Alternative_allele	Effect_allele	P_value	Consequences_annotation	Nearest_genes
1	rs61783804	28717871	C	T	T	4.23E-09	intronic	*PHACTR4*
1	rs7515828	73848331	T	C	C	6.29E-16	intergenic	*LINC01360(dist=43771),LRRIQ3(dist=643371)*
2	rs1004787	45159091	G	A	A	1.79E-11	intergenic	*CAMKMT(dist=159360),SIX3-AS1(dist=8202)*
2	rs1474011	146118069	G	A	A	7.98E-13	intergenic	*TEX41(dist=283778),PABPC1P2(dist=1226556)*
7	rs6969783	117593308	A	T	T	2.48E-10	intergenic	*CTTNBP2(dist=79747),LSM8(dist=230778)*
8	rs2565060	27336767	T	A	A	6.04E-14	UTR5	*CHRNA2(NM_000742:c.-8192T>A,NM_001282455:c.-8192T>A)*
9	rs112270518	136471660	A	G	G	1.96E-09	intergenic	*FAM163B(dist=26292),DBH(dist=29825)*
10	rs11191512	104772699	G	A	A	3.55E-09	intronic	*CNNM2*
11	rs7126748	112842976	C	T	T	8.15E-12	intronic	*NCAM1*
11	rs3133388	113407114	A	G	G	1.12E-11	intergenic	*DRD2(dist=61113),TMPRSS5(dist=151154)*
12	rs6580707	49763868	A	G	G	3.88E-09	intronic	*SPATS2*
15	rs28505872	47844059	T	C	C	4.28E-08	intronic	*SEMA6D*
16	rs7500367	50939192	C	G	G	2.81E-08	intergenic	*CYLD(dist=103346),LOC101927334(dist=112477)*
16	rs4985379	69949196	C	T	T	1.65E-08	intronic	*WWP2*
18	rs154908	58839676	C	T	T	3.81E-08	intergenic	*MC4R(dist=799675),CDH20(dist=318099)*
19	rs12459249	41339896	T	C	C	1.50E-13	intergenic	*RAB4B-EGLN2(dist=25550),CYP2A6(dist=9547)*

Supplementary Table 2. Genome-wide significant associations for cigarette per day (CPD) in the European American samples in the Million Veteran Program

Phenotype	Chromosome	SNP	Position	Reference_allele	Alternative_allele	Effect_allele	Beta	Test_statistic	P_value	Consequences_annotation	Nearest_genes	P_value_adjusted
Cigarette per day current	9	rs3025386	136502764	T	C	C	-0.017	-5.694	1.25E-08	intronic	*DBH*	1.42E-08
	15	rs12914385	78898723	C	T	T	0.014	6.243	4.30E-10	intronic	*CHRNA3*	5.00E-10
	20	rs11697662	61992005	C	T	C	0.016	5.793	6.93E-09	ncRNA_intronic	*LOC100130587*	7.89E-09
Cigarette per day past	2	rs7571606	146123058	T	A	A	0.027	5.861	4.62E-09	intergenic	*TEX41(dist=288767),PABPC1P2(dist=1221567)*	6.26E-09
	15	rs8040868	78911181	T	C	C	0.044	9.274	1.80E-20	exonic	*CHRNA3*	3.80E-20
	19	rs12459249	41339896	T	C	T	-0.033	-6.766	1.33E-11	intergenic	*RAB4B-EGLN2(dist=25550),CYP2A6(dist=9547)*	1.99E-11

P_value_adjusted is the P-value derived from the adjusted test statistic, which is the ratio between the test statistic and the intercept from the LD score regression. P-values that reached genome-wide significance (<5E-08) after the adjustment were colored in red.

Supplementary Table 3. SNP-based heritability and inflations for smoking phenotypes in European American samples in the Million Veteran Program estimated from the LD score regression

MVP EA Phenotype	Heritability	Standard_error	Genomic inflation factor	LD score regression intercept with standard error
Trajectory contrast I (current vs. never)	18.7%	0.010	1.344	1.0281 (0.0096)
Trajectory contrast II (current vs. mixed)	5.8%	0.005	1.181	1.0236 (0.0091)
Smoking initiation (ever vs. never)	6.9%	0.004	1.198	1.0125 (0.0084)
Smoking cessation (current vs. past)	6.1%	0.005	1.197	1.0580 (0.0094)

EA: European American
