# Supplementary material for: Genome-wide association study of smoking trajectory and meta-analysis of smoking status in 842,000 individuals
Source: Nat Commun. 2020 Oct 20;11:5302. doi: 10.1038/s41467-020-18489-3 (PMC7598939; doi:10.1038/s41467-020-18489-3)
Supplement: Supplementary file 2 — Descriptions of Additional Supplementary Files [file 41467_2020_18489_MOESM2_ESM.pdf]

Descriptions of Additional Supplementary Files

Supplementary Data 1.

Description: Genome-wide significant associations for smoking phenpotypes in the Million Veteran Program (N=286,118).

Supplementary Data 2.

Description: Genome-wide significant associations for a trans-ethnic meta analysis of smoking trajectories in European Americans, African Americans, and Hispanic Americans in the Million Veteran Program.

Supplementary Data 3.

Description: Genome-wide significant associations for a meta analysis of smoking status in European American (EA) samples in the Million Veteran Program (MVP) and European samples from the GWAS & Sequencing Consortium of Alcohol and Nicotine use (GSCAN excluding 23andMe.

Supplementary Data 4.

Description: Heritability enrichment for smoking phenotypes in the European American samples in the Million Veteran Program.

Supplementary Data 5.

Description: Gene prioritization for smoking trajectories in the European American samples in the Million Veteran Program (MVP) using functional mapping and annotation (FUMA).

Supplementary Data 6.

Description: Gene enrichment analysis for smoking trajectories in the European American samples in the Million Veteran Program (MVP).

Supplementary Data 7.

Description: Gene correlation analysis for smoking trajectory contrasts in the European American samples in the Million Veteran Program.

Supplementary Data 8.

Description: Mendelian randomization (MR) analysis for smoking trajectory contrasts in the European American samples in the Million Veteran Program.
