# Supplementary material for: Genome-wide association study of smoking trajectory and meta-analysis of smoking status in 842,000 individuals
Source: Nat Commun. 2020 Oct 20;11:5302. doi: 10.1038/s41467-020-18489-3 (PMC7598939; doi:10.1038/s41467-020-18489-3)
Supplement: Supplementary file 11 — Reporting Summary [file 41467_2020_18489_MOESM11_ESM.pdf]

nature research

Corresponding author(s): Amy Justice

Last updated by author(s): Jul 3, 2020

Reporting Summary

Nature Research wishes to improve the reproducibility of the work that we publish. This form provides structure for consistency and transparency in reporting. For further information on Nature Research policies, see our Editorial Policies and the Editorial Policy Checklist.

Statistics

For all statistical analyses, confirm that the following items are present in the figure legend, table legend, main text, or Methods section.

n/a	Confirmed	
[ ]	[x]	The exact sample size (*n*) for each experimental group/condition, given as a discrete number and unit of measurement
[ ]	[x]	A statement on whether measurements were taken from distinct samples or whether the same sample was measured repeatedly
[ ]	[x]	The statistical test(s) used AND whether they are one- or two-sided<br>*Only common tests should be described solely by name; describe more complex techniques in the Methods section.*
[ ]	[x]	A description of all covariates tested
[ ]	[x]	A description of any assumptions or corrections, such as tests of normality and adjustment for multiple comparisons
[ ]	[x]	A full description of the statistical parameters including central tendency (e.g. means) or other basic estimates (e.g. regression coefficient) AND variation (e.g. standard deviation) or associated estimates of uncertainty (e.g. confidence intervals)
[ ]	[x]	For null hypothesis testing, the test statistic (e.g. *F*, *t*, *r*) with confidence intervals, effect sizes, degrees of freedom and *P* value noted<br>*Give P values as exact values whenever suitable.*
[x]	[ ]	For Bayesian analysis, information on the choice of priors and Markov chain Monte Carlo settings
[x]	[ ]	For hierarchical and complex designs, identification of the appropriate level for tests and full reporting of outcomes
[ ]	[x]	Estimates of effect sizes (e.g. Cohen's *d*, Pearson's *r*), indicating how they were calculated

Our web collection on statistics for biologists contains articles on many of the points above.

Software and code

Policy information about availability of computer code

Data collection	Phenotypes were extracted from electronic medical records and responses to survey questions. Affymetrix Axiom Biobank Array was used for genotyping and Minimac3 for genotype imputation.
Data analysis	We used PLINK (v1.9) for association test (logistic/linear), SNPTEST (v2.5.4-beta2) for association test (multinomial), flashpca (v1.2.5) to perform principal component analysis for population structure, METAL (v2011-03-25) for meta-analysis of GWAS results, ANNOVAR (v2016Feb01) to map index SNPs to their nearest genes, LocusZoom (v1.3) to visualize regional associations, functional mapping and annotation tool (FUMA, v1.3.6) for gene prioritization, LD score regression (v1.0.0) and GenoSkyline-Plus (v1.0.0) functional annotations for heritability estimation and heritability enrichment analysis, GeNetic cOVariance Analyzer (GNOVA) for genetic correlation analysis, DAVID (v6.8) for pathway enrichment analysis, and R packages MendelianRandomization (v0.4.2) and MRPRESSO (v1.0) to perform mendelian randomization to identify potential causal-consequential relationships between complex traits.

For manuscripts utilizing custom algorithms or software that are central to the research but not yet described in published literature, software must be made available to editors and reviewers. We strongly encourage code deposition in a community repository (e.g. GitHub). See the Nature Research guidelines for submitting code & software for further information.

Data

Policy information about availability of data

All manuscripts must include a data availability statement. This statement should provide the following information, where applicable:

- Accession codes, unique identifiers, or web links for publicly available datasets
- A list of figures that have associated raw data
- A description of any restrictions on data availability

The full summary-level association data from the meta-analysis for each of the smoking-related traits from this report are available through dbGaP accession

number phs001672.v1.p1 [https://www.ncbi.nlm.nih.gov/projects/gap/cgi-bin/study.cgi?study_id=phs001672.v1.p1]. 1000 Genomes Project reference panel can be downloaded from [ftp://ftp.1000genomes.ebi.ac.uk/vol1/ftp/]. GWAS summary statistics used in the genetic correlation analysis were made publicly available by GSCAN [https://conservancy.umn.edu/handle/11299/201564], Social Science Genetic Association Consortium (SSGAC) [https://www.thessgac.org/data], and LD Hub [http://ldsc.broadinstitute.org/ldhub].

Field-specific reporting

Please select the one below that is the best fit for your research. If you are not sure, read the appropriate sections before making your selection.

- [x] Life sciences
- [ ] Behavioural & social sciences
- [ ] Ecological, evolutionary & environmental sciences

For a reference copy of the document with all sections, see nature.com/documents/nr-reporting-summary-flat.pdf

Life sciences study design

All studies must disclose on these points even when the disclosure is negative.

Sample size	We defined ancestral population using genotypes of individuals from the Million Veteran Program and 1000 Genomes Project. We applied a series quality control steps detailed in the Methods section. A total of 209,915 European Americans, 54,867 African Americans, and 21,336 Hispanic Americans passed quality control filters. Using the 25% prevalence of smoking in the general population, the statistical power to detect variants with a 5% of minor allele frequency using an additive genetic association model and genome-wide significance threshold of p < 5E-8 is 99% for EAs and 96% for AAs. Our study cohort is well-powered to identify genetic associations.
Data exclusions	We followed Anderson et al. (Nature Protocol, 2010) and performed quality control on both study participants and genetic variants. We removed samples with a high genotype missing rate (>10%), discordant sex, excessive heterozygosity (>3 standard deviation), and up to second-degree relatives. For genetic variations, we filtered out rare variants (minor allele frequency < 0.01), variants with a missing rate > 5%, variants with imputation r-squared < 0.8, and those that deviated significantly from Hardy-Weinberg equilibrium (p<1x10-6). The exclusion criteria were established before the study.
Replication	We identified genetic markers significantly associated with smoking behaviors in the Million Veteran Project cohort and compared the identified associated genes to previous publications including the largest smoking GWAS to date conducted by GSCAN. We summarized the replication results in Supplementary Table 2 and Supplementary Table 5. We were able to replicate a subset of loci identified in our study cohort. Some other genetic associations cannot be replicated potentially due to the fact that our smoking phenotypes were derived from longitudinal data points from the electronic health record (EHR) whereas the phenotypes in GSCAN were based on one-time self-reported survey data.
Randomization	Smoking is an observational behavior. Randomization is not applicable.
Blinding	Genotyping was done blind to phenotypes.

Reporting for specific materials, systems and methods

We require information from authors about some types of materials, experimental systems and methods used in many studies. Here, indicate whether each material, system or method listed is relevant to your study. If you are not sure if a list item applies to your research, read the appropriate section before selecting a response.

Materials & experimental systems

n/a	Involved in the study	
x		Antibodies
x		Eukaryotic cell lines
x		Palaeontology and archaeology
x		Animals and other organisms
	x	Human research participants
x		Clinical data
x		Dual use research of concern

Methods

n/a	Involved in the study	
x		ChIP-seq
x		Flow cytometry
x		MRI-based neuroimaging

Human research participants

Policy information about studies involving human research participants

Population characteristics	Our study cohort is a multi-ethnic cohort with 209,915 European Americans (73% EAs), 54,867 African Americans (19% AAs), and 21,336 Hispanic Americans (8% HAs). The mean age of the study cohort ranged from 58 to 64 years across three ancestral groups (EA: mean=64, standard deviation (SD)=13, AA: mean=58, SD=12, and HA: mean=56, SD=15) and majority of the MVP samples were male (EA: 93%, AA: 87%, and HA: 92%). All individuals had their genotype data available. Smoking data from 2000-2015 were collected from participants during medical visits and the mean number of the medical visits is 8 times. Based on the final designation of smoking trajectory groups, we identified 56,887 (20%) mostly current smokers, 145,229 (51%) mixed smokers, and 84,002 (29%) mostly never smokers.
Recruitment	The Recruitment began in 2011 with ~50 recruiting sites nationwide. Recruitment is conduced in person at selected sites in the VHA health care system. Every Veteran is assigned a study ID number that links phenotype, genotype, and clinical information. Given the veteran population, most participants are male in the senior age group. Based on self-reported race, white (77.2%) and African American (13.5%) populations are well represented. Overall, our findings generally apply to the senior male population but not to the under-represent groups (female, younger age group, Hispanic Americans and other minority race/ethnic groups).
Ethics oversight	The central Veteran Affairs Institutional Review Board (IRB) and site-specific IRBs approved the MVP study. All relevant ethical regulations for work with human subjects were followed in the conduct of the study and informed consent was obtained from all participants.

Note that full information on the approval of the study protocol must also be provided in the manuscript.
